# Medical Opinion and Movement

**Published:** 1907-01-05

**Authors:** 


### MEDICAL OPINION AND MOVEMENT.

THE Odontological Society of Great Britain is prepared to receive applications for grants in aid of the furtherance of scientific research in connection with dentistry. The grants expire on May 1 next following the date on which they are made. A report of the work must be furnished to the Committee of the Society by the recipient of the grant on April 20 following the allotment; and in the event of the whole expense of the inquiry being provided by the Committee, the result of the investigation shall be the property of the Society. In any case, the author shall not publish a report of the work done elsewhere than in the Transactions of the Society, except by permission of the Committee. Further particulars may be obtained on application to the Hon. Secretary, Scientific Research Committee, Odontological Society, 20 Hanover Square, W.

THE Courts of Law afford abundant evidence of the unwarrantable attacks to which members of the medical profession are continually exposed in the shape of allegations of improper treatment by dissatisfied patients or by the slanderous assertions of the hysterical. It is incumbent on the medical man to give his full attention to the exercise of his professional duties, and to display all that sympathy and kindliness which the condition of his patient naturally calls forth, but he must be careful to be wary for himself and the consequences which may befall him should he fail in any way to take such precautions as would preclude the possibility of his falling into the entanglement of "legal proceedings." A rather extreme case has recently been decided by the Supreme Court of Michigan. A lad, aged seventeen, consulted a surgeon for a small tumour of the ear. At the time of his visit he was accompanied by several adult relatives—an aunt and two sisters. He submitted to an operation, and died during the administration of the anæsthetic. The father sued the doctors on the ground that his consent had not been asked. The Court naturally held that, in view of the absence of any reasons to lead the surgeon to believe that the consent of the relatives did not include that of the father, no liability was incurred by the medical men concerned.

THERE has been for some time a general consensus of opinion that the teaching of midwifery in the medical schools is inadequate, and some time ago a Committee was appointed by the General Medical Council to investigate the question and devise means of improvement. Some of the recommendations of the Committee are already carried out in most of the London schools of medicine, and there should be little difficulty in making them compulsory for all. Such is, for instance, the recommendation that the student shall not be allowed to conduct his twenty cases of labour before he has filled the post of clinical clerk and surgical dresser and has attended a course of lectures in surgery, medicine, and midwifery. But the Committee makes a further recommendation, that every student, before attending his twenty cases of labour, must have attended a lying-in hospital for three months, or during one month have given daily attendance at a lying-in hospital or in the lying-in wards of a general hospital, and therein conducted cases of labour under the personal supervision of a medical officer of the hospital. The majority of the hospitals have unhesitatingly condemned this proposal as impracticable. Its enforcement would undoubtedly seriously handicap those hospitals which are not provided with special obstetric wards, and would add further outlay to an already expensive curriculum. There can, however, be no two opinions that the present manner in which the medical student acquires his obstetric experience and knowledge is altogether too haphazard to be efficient, and if the Committee is convinced of the necessity for this provision, means should be found to give it practical effect. It is of the greatest importance that the general practitioner should commence his professional career with a sound, practical knowledge of midwifery.

WITH the Education Bill defunct, the provision for medical inspection of the children of public elementary schools must necessarily for the time being remain in abeyance. It is at least a step in the right direction that the principle of the provision received such general support from the various political parties, but it is to be hoped that in the event of a still further protracted delay in the settlement of the education controversy some means may be found of giving practical effect to this part of the Bill. We are accustomed to regard ourselves as a nation in the foremost van of civilisation, yet we are strangely halting in our progress in these matters. In many parts of Germany the education authorities not only provide for the medical inspection of all school children, but have also called into requisition the services of the dental surgeon. Proper supervision of the children's teeth has been carried out in Strasburg since 1902 by a dental surgeon duly appointed by the city, and the German dental societies are now making strenuous efforts to force upon the attention of the authorities the importance of the proper care of the teeth, and to induce them to carry out the recommendations of the Congress of the International Dental Federation, held in August 1906. These recommendations were that the children should be taught the proper care of their teeth and the importance of a healthy, clean mouth, also that school clinics should be established where the children's teeth might receive the necessary attention and supervision. The medical profession is now fully alive to the fact that carious teeth are responsible for many disorders, often of a serious nature, and it is recognised that a healthy mouth is one of the chief essentials for a healthy body. Any medical supervision of school children, therefore, which does not include also due attention to the condition of the teeth will be defective and unsatisfactory. It is only by bringing this aspect of the matter before the notice of the authorities that we can hope to see adequate dental inspection enforced.

